# Metabolomics assisted by transcriptomics analysis to reveal metabolic characteristics and potential biomarkers of early-onset colorectal cancer

**DOI:** 10.1007/s12672-026-05185-9

**Published:** 2026-05-28

**Authors:** Hui Zhang, Jiangping Yu, Rongwei Ruan, Zhao Cui, Yali Tao, Shuwen Zhu, Shi Wang

**Affiliations:** Department of Endoscopy, Zhejiang Cancer Hospital, Hangzhou Institute of Medicine (HIM), Chinese Academy of Sciences, Banshan Road 1, Gongshu District, Hangzhou, 310022 Zhejiang China

**Keywords:** Early-onset colorectal cancer, LC-MS, Biomarker, Metabolomics, Transcriptomics

## Abstract

**Background:**

Metabolic reprogramming plays a crucial role in the occurrence and progression of colorectal cancer (CRC). However, the relationship between metabolism and CRC development remains to be fully elucidated.

**Methods:**

We collected 45 colorectal tissue samples, including 15 cases of early-onset colorectal cancer (EOCRC), 15 cases of later-onset colorectal cancer (LOCRC), and 15 adjacent normal tissues for metabolomic analysis. A prediction model based on differentially expressed metabolites (DEMs) was established using the random forest algorithm with 10-fold cross-validation. Transcriptome analysis was performed on the GSE39084 dataset, which included 25 EOCRC and 35 LOCRC cases. Pathway-level integrative analyses combining metabolomics and transcriptomics were further conducted.

**Results:**

Significant differences in metabolic profiles were observed among EOCRC, LOCRC, and adjacent normal tissues. A total of 141 DEMs were identified, showing significant enrichment in 20 metabolic pathways. The random forest model, consisting of 10 metabolites, achieved a cross-validated area under the curve (AUC) value of 0.916 for distinguishing EOCRC from other groups. Weighted Gene Co-expression Network Analysis (WGCNA) revealed a gene module strongly positively correlated with Lynch syndrome, microsatellite instability (MSI) status, and PIK3CA mutations. Pathway-level integrative analysis highlighted the Citrate cycle (TCA cycle) and Central carbon metabolism pathways as consistently enriched at both metabolic and transcriptional levels. Furthermore, four metabolism-related genes were identified as candidate genes warranting further investigation in EOCRC.

**Conclusion:**

The integration of metabolomics and transcriptomics in this study provides novel insights into the pathological alterations associated with EOCRC, facilitating the identification of candidate therapeutic targets.

## Introduction

Colorectal cancer (CRC) is the third most prevalent malignancy and the second leading cause of mortality globally [1]. In recent years, advancements in early screening, monitoring, and treatment have contributed to a decline in CRC incidence and mortality rates. However, there has been an alarming rise in the occurrence and fatality of early-onset colorectal cancer (EOCRC), defined as colorectal cancer diagnosed before the age of 50 [2–5]. EOCRC exhibits characteristics such as later-stage diagnosis, poorer prognosis based on histological classification, and greater molecular spectrum variation compared to later-onset colorectal cancer (LOCRC) occurring at or after 50 years old. Currently, conventional treatment methods for CRC are predominantly employed for EOCRC patients with limited targeted therapeutic options available [6, 7]. Enhancing early detection rates among EOCRC patients and identifying potential targets for early diagnosis and treatment are pressing issues that demand immediate attention.

Reprogrammed metabolism is a crucial manifestation that supports tumor growth, serving as both a target for tumor treatment and a potential resource for studying tumor biomarkers [8, 9]. Reprogrammed metabolism represents a distinctive characteristic of colorectal cancer (CRC), where CRC cells are specifically adapted to rapid proliferation in nutrient-poor environments, necessitating the acquisition of nutrients and efficient removal of cellular waste [10].

The etiology of EOCRC is multifactorial and may result from interactions between genetic variants and environmental risk factors. For instance, obesity, diabetes, smoking, and alcohol consumption are well-documented lifestyle factors associated with EOCRC [2]. Furthermore, dietary factors such as high-fat diet (HFD) are strongly associated with the development of EOCRC [11]. Metabolomics approaches are emerging strategies for identifying and treating EOCRC. However, the abnormal metabolic regulation in EOCRC remains to be elucidated. The techniques for metabolomics analysis including nuclear magnetic resonance spectroscopy (NMR), gas chromatography-mass spectrometry (GC-MS), capillary electrophoresis-mass spectrometry (CE-MS), and liquid chromatography-mass spectrometry (LC-MS), have been increasingly utilized for profiling metabolic features of various pathologies in numerous cancers, including CRC [12–15]. Despite the presence of multi-omics studies on EOCRC, limited attention has been devoted to the early diagnosis of EOCRC [16, 17]. Furthermore, to our knowledge, these studies have solely relied on plasma samples and fail to fully elucidate the intrinsic mechanism underlying EOCRC. Therefore, it remains highly significant to integrate multiple testing approaches in order to enhance the early detection rate among EOCRC patients and identify potential targets for treatment.

In our study, untargeted LC-MS metabolic profiling was employed to characterize the metabolites in EOCRC, facilitating the detection and identification of potential dysregulated expressed metabolites (DEMs) and discovery of metabolic markers. Additionally, transcriptome analysis based on GEO databases was conducted to identify differentially expressed genes (DEGs) between EOCRC patients and LOCRC patients. By jointly analyzing metabolomic and transcriptomic data at the pathway level, significantly altered metabolic pathways associated with EOCRC were identified. The objective of this study was to explore potential biomarkers and elucidate relevant metabolic pathways linked to EOCRC. Furthermore, it holds significant potential for acquiring novel insights into the pathological alterations associated with EOCRC, thereby facilitating the identification of new therapeutic targets.

## Materials and methods

### Sample collection

The EOCRC, LOCRC, and paracancerous tissues were collected at Zhejiang Cancer Hospital. They were promptly preserved in liquid nitrogen following surgical resection and subsequently stored in a cryogenic freezer at −80 degrees Celsius. Prior to the surgical resection, patients did not undergo any radiotherapy or chemotherapy.

### Liquid chromatography-mass spectrometry (LC/MS) analysis

The metabolomic analysis of CRC and normal tissue was conducted using liquid chromatography and mass spectrometry (LC-MS) metabolomic analysis. Subsequently, the data were converted to mzXML format using Proteowizard software and processed using the R package (version 4.4). For multivariate analysis, the orthogonal partial least squares discriminant analysis (OPLS-DA) methods were employed. Permutation tests (200 permutations) were performed to assess model overfitting and robustness. Furthermore, differentially expressed metabolites (DEMs) were identified based on a threshold of *P* value ≤ 0.05 and a fold-change threshold, with a false discovery rate (FDR) correction (Benjamini-Hochberg) applied post-hoc; key findings were robust to an FDR < 0.1. Metabolite annotation was performed by matching accurate mass, retention time, and MS/MS spectra against multiple databases, including MassBank, mzCloud, LipidMaps, METLIN, and the Human Metabolome Database, as well as a self-built standards database at Personal Biotechnology Co., Ltd. (Shanghai, China). Because this was an untargeted LC-MS study, the confidence of metabolite identification varied across compounds. Some metabolites were supported by reference standards or high-quality MS/MS spectral matching, whereas others should be considered putatively annotated, particularly in cases where isomeric compounds could not be unequivocally distinguished [18, 19]. Therefore, downstream biological interpretation and pathway enrichment results were interpreted with appropriate caution. Additionally, the metabolic pathway information of DEMs was revealed through KEGG in MetaboAnalyst (version 6.0, http://www.metaboanalyst.ca/) [20].

### Transcriptomic analysis

The GSE39084 dataset, which consists of 25 EOCRC cases and 35 LOCRC cases, was selected from the Gene Expression Omnibus (GEO) database for transcriptome analysis. Differential expression analysis was conducted using DESeq2 with a negative binomial distribution (NB) test. Significantly differentially expressed genes (DEGs) were identified based on a threshold of adjusted P value (FDR) < 0.05. To illustrate the expression pattern of genes in different groups and samples, hierarchical cluster analysis of DEGs was performed using R (version 4.4).

### Weighted correlation network analysis (WGCNA)

WGCNA was used to further identify key modules associated with EOCRC, which can identify highly synergistic gene modules and candidate biomarkers based on the intrinsic connections of gene networks [21]. The gene co-expression network was constructed by selecting the DEGs matrix in GSE39084 dataset. The minimum number of module genes was defined as 40. Modules were evaluated for association with clinicopathological features including age (treated as a continuous variable in the correlation matrix), sex, Lynch syndrome, tumor location, ajcc TNM stage, Microsatellite Instability (MSI), CpG Island Methylator Phenotype (CIMP), TP53, Kras, Braf, and PIK3CA.

### Enrichment analysis

Regarding the transcriptional data, Gene Ontology (GO) and KEGG pathway enrichment analysis of DEGs were performed, and R (version 4.4) was used to screen significantly enriched terms, respectively. Co-expression network of metabolites and genes was analyzed by the metaboanalyst database (https://www.metaboanalyst.ca/).

### Integrative analysis of metabolome and transcriptome

The integrated analysis of transcriptome and untargeted metabolomics was conducted as a joint pathway enrichment analysis, focusing on the overlap of significantly enriched pathways from both datasets rather than direct gene–metabolite correlations across matched samples. Because the metabolomic and transcriptomic data were derived from independent cohorts, the results should be interpreted as pathway-level convergence rather than individual-level multi-omics associations. All DEGs and DEMs were mapped to the MetaboAnalyst database for pathway-level integrative analysis and network visualization.

### Statistical analysis

The R 4.4 software was employed for comprehensive data processing, statistical analysis, and mapping. Post-hoc power analysis was performed using the pwr package; based on observed effect sizes (Cohen’s d > 1.5), a sample size of 15 per group provided > 80% power at α = 0.05. Spearman correlation coefficients were utilized to evaluate the associations among continuous variables. The chi-square test was used to compare categorical variables, while the Wilcoxon rank-sum test or T-test was applied for comparing continuous variables. The survminer software package was utilized to determine the optimal cut-off value. We used the CompareC software package to compare variable-specific c-indices. For predicting binary categorical variables, the receiver operating characteristic curve (ROC) was generated using the pROC package, and 95% confidence intervals for AUC values were calculated. The random forest diagnostic model was built using 10-fold cross-validation with 10 repeats to mitigate overfitting; the reported AUC is the mean cross-validated AUC. Time-dependent area under the ROC curve (AUC) analysis of survival variables was performed using timeROC software package. All statistical tests adopted a two-sided with a significance level set at *P* < 0.05.

## Results

### Screening and identification of differential metabolites in EOCRC, LOCRC, and adjacent normal tissues using LC-MS metabolomic analysis

**Fig. 1:**
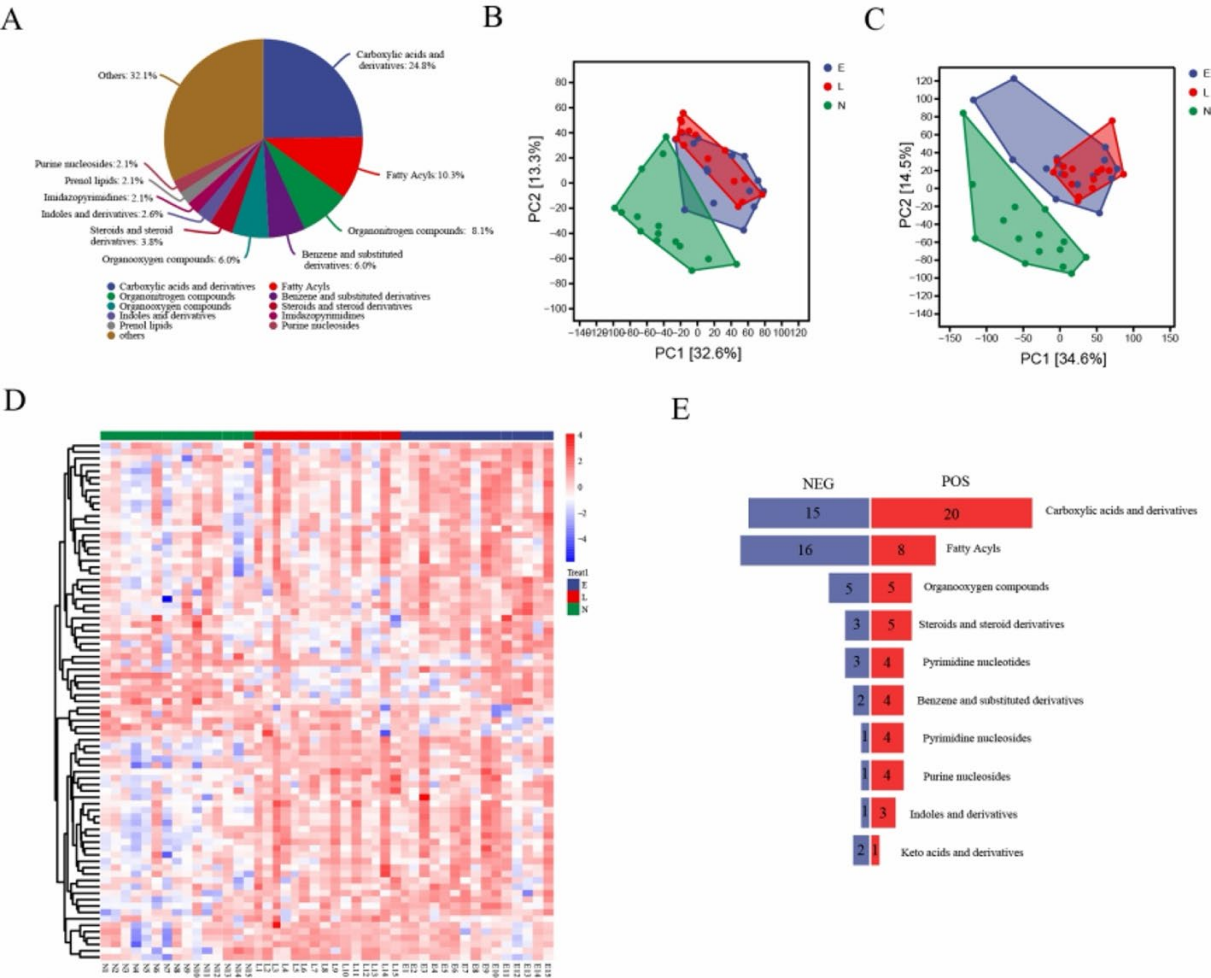
Screening and identification of DEMs in EOCRC, LOCRC, and adjacent normal tissues using LC-MS metabolomic analysis. **A** The main metabolites screened by LC-MS analysis. **B** The OPLS-DA plot for the NEG data from EOCRC, LOCRC and normal tissue. **C** The OPLS-DA plot for the POS data from EOCRC, LOCRC and normal tissue. **D** Heatmap of DEMs from individual comparisons among different class. **E** The distribution of the counts of DEMs among the NEG group and POS group

The metabolic profiles of 15 cases of EOCRC, 15 cases of LOCRC, and 15 cases of adjacent normal tissues were analyzed using LC-MS to investigate the metabolic alterations in CRC. The top three metabolite categories were Carboxylic acids and derivatives (24.8%), Fatty Acyls (10.3%), and Organonitrogen compounds (8.1%), as depicted in Fig. 1A. The Partial Least Squares Discriminant Analysis (PLS-DA) was employed to examine the distinctions among three groups. The PLS-DA score scatter plots of positive ion (POS) and negative ion (NEG) were displayed in Fig. 1B and C. The observed trend of segregating normal tissues from EOCRC and LOCRC tissues suggests distinct metabolite levels between CRC and normal tissues. Permutation testing (200 permutations) confirmed that the OPLS-DA models were not overfitted (*P* < 0.05). In order to comprehensively elucidate the association between EOCRC, LOCRC, and adjacent normal samples, hierarchical clustering analysis was conducted based on the expression profiles of all significantly different metabolites. As depicted in Fig. 1D, a total of 141 differentially expressed metabolites (DEMs) were identified among the three groups. Figure 1E further illustrates the distribution pattern of these DEMs in the POS and NEG groups. Notably, within the set of 141 DEMs, there were 35 Carboxylic acids and derivatives as well as 24 Fatty Acyls.

### KEGG pathway related to EOCRC

**Fig. 2:**
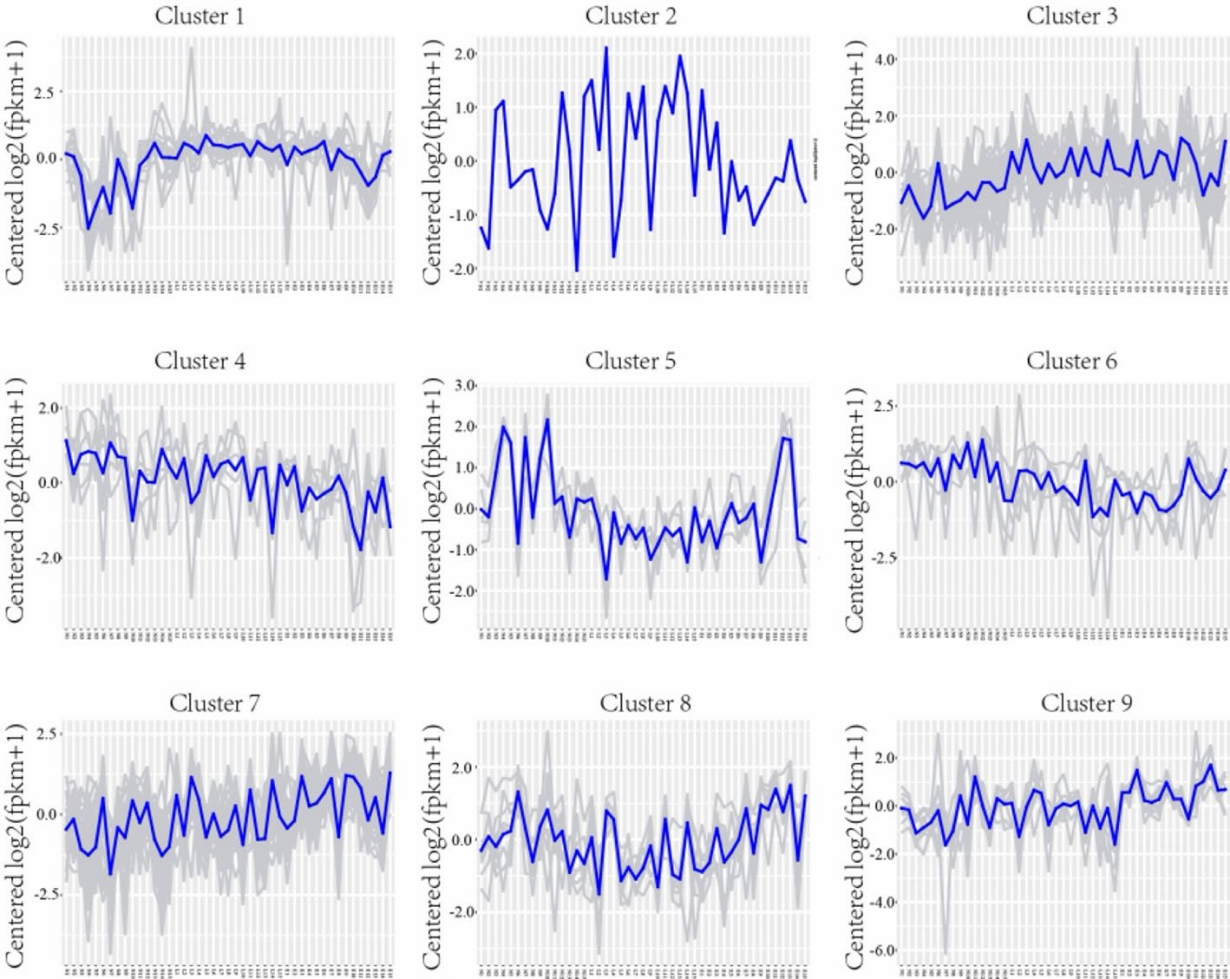
The trend analysis of differential metabolites. The gray line in the figure shows the expression pattern of metabolites in each cluster, and the blue line represents the average expression of all metabolites in the cluster of the all samples

**Fig. 3:**
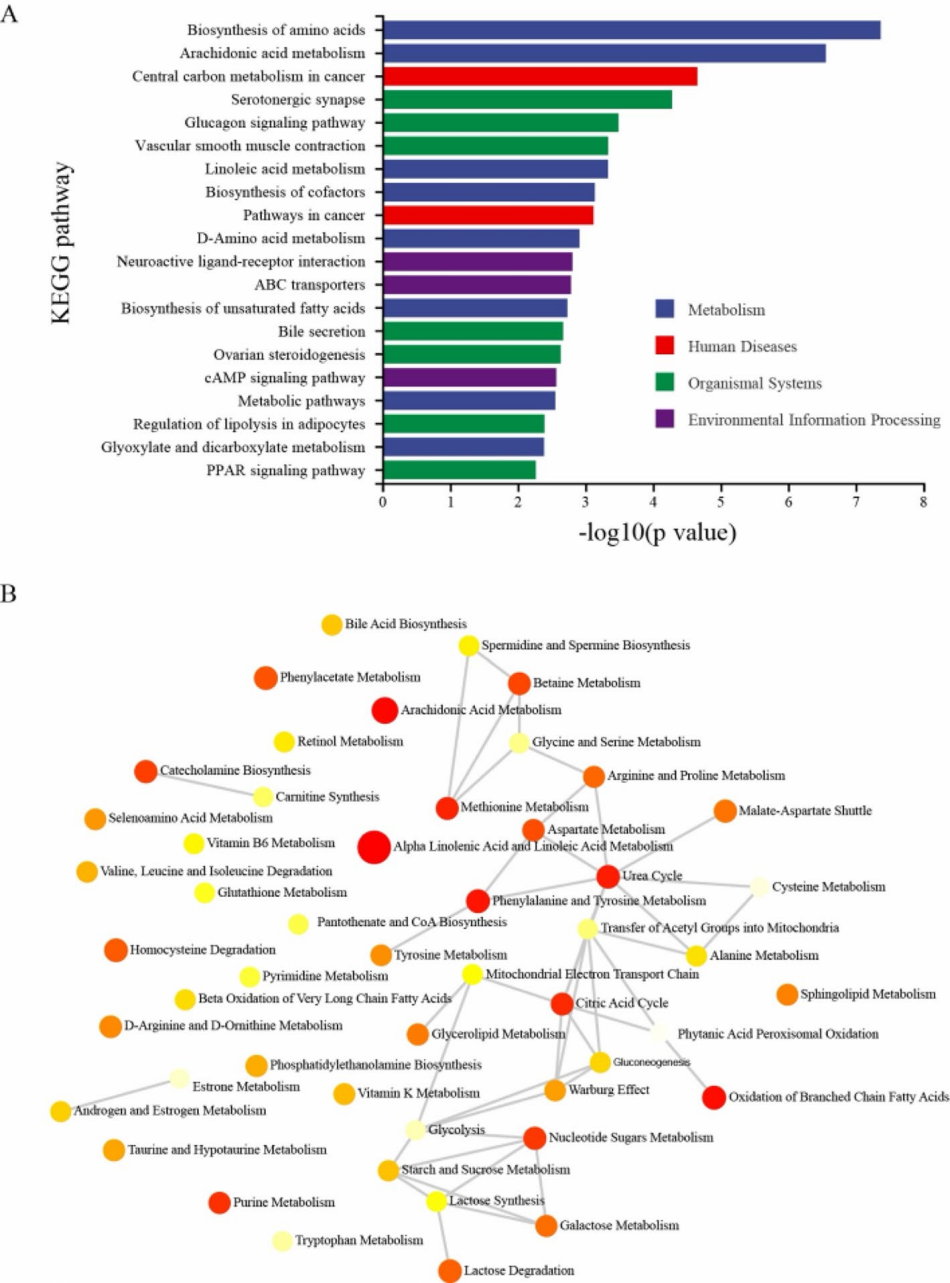
KEGG pathway enrichment analysis and pathway network were performed based on DEMs. **A** The top 20 metabolic pathways with the highest enrichment were determined based on DEMs. **B** Network analysis of metabolic pathway was performed by the MetaboAnalyst database

The total 141 DEMs were split into 9 groups (Fig. 2) based on their trend similarity over time using the soft Mfuzz clustering algorithm. To further elucidate and comprehend the biological significance of those DEMs among the EOCRC group, LOCRC group, and normal tissue group, KEGG enrichment analysis was conducted to identify the most significant metabolic pathways (Fig. 3A). The top three enriched pathways included Biosynthesis of amino acids, Arachidonic acid metabolism and Central carbon metabolism in cancer. The network diagram in Fig. 3B depicted the interconnections among multiple metabolic pathways. The above results suggest that these altered metabolites and pathways may be associated with EOCRC.

### Comprehensive analyses of 10 metabolites using random forest analysis

**Fig. 4:**
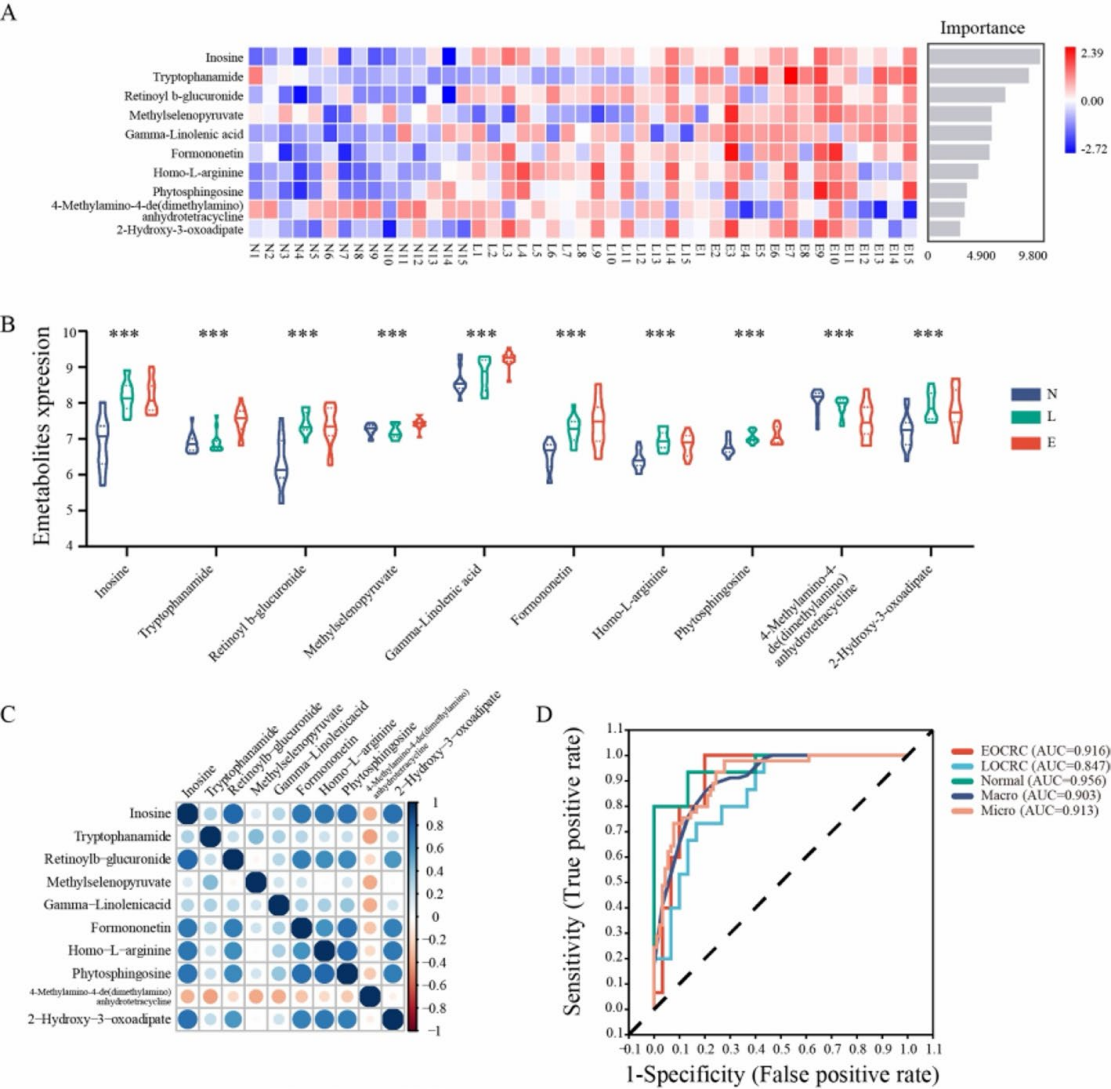
Comprehensive analyses of 10 metabolites using random forest analysis. **A** The top 10 metabolites were selected by the random forest. **B** Violin plot depicts the expression of 10 metabolites in EOCRC, LOCRC and normal tissues. **C** Spearman correlation analysis among the 10 metabolites. **D** The ROC curve was utilized to assess the effectiveness of a prognostic model consisting of 10 metabolites in distinguishing EOCRC, LOCRC, and normal tissues. ***P < 0.001

We performed a random forest analysis based on these 141 DEMs to identify the key metabolites. The heat map visualized the distribution of relative abundance for the top 10 most significant metabolites (Fig. 4A), while their expression among EOCRC, LOCRC, and normal tissues was depicted in Fig. 4B. The correlation heatmap in Fig. 4C revealed significant positive correlations among Inosine, Retinoyl b-glucuronide, Formononetin, Homo-L-arginine, Phytosphingosine, and 2-Hydroxy-3-oxoadipate. Conversely, these variables exhibited negative correlations with 4-Methylamino-4-de(dimethylamino). As depicted in Fig. 4D, the levels of these ten metabolites were utilized to construct an OPLS-DA model, which demonstrated remarkable efficacy in discriminating between EOCRC patients with LOCRC and normal tissue. The random forest model demonstrated promising discriminative performance, with a mean cross-validated AUC of 0.916 (95% CI: 0.85–0.97) for EOCRC, 0.847 for LOCRC, and 0.956 for normal tissue across all samples. However, given the relatively small sample size and single-center study design, these results should be considered preliminary and interpreted with caution.

### The DEGs screened by WGCNA analysis

**Fig. 5:**
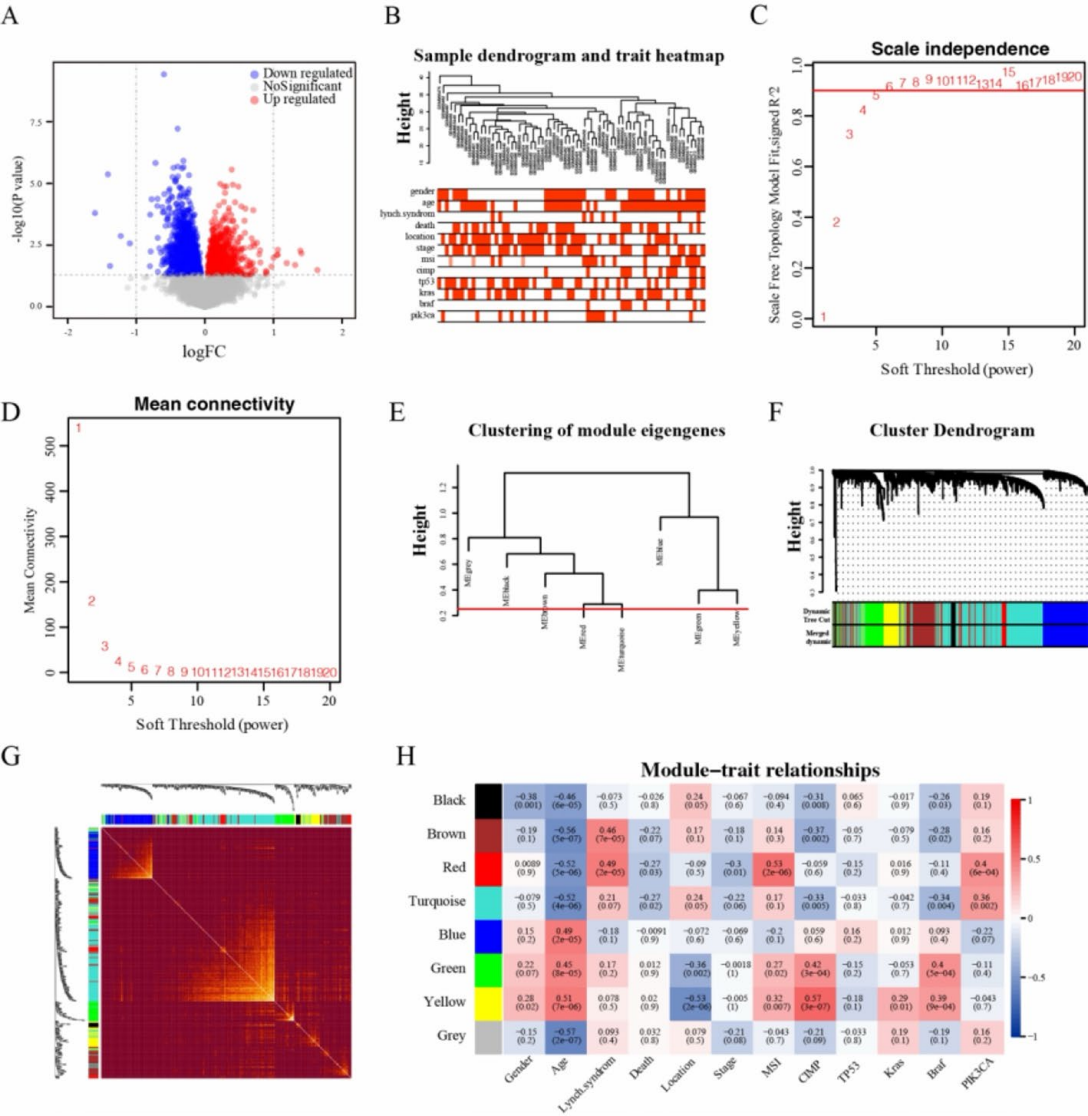
WGCNA analysis screened the key gene modules. **A** volcano plot of DEGs between EOCRC and LOCRC groups. Red indicates significantly increased genes. Blue indicates significantly decreased genes. **B** Clustering dendrogram of 70 samples. **C** Analysis of the scale-free index for various soft-threshold powers (β). **D** Analysis of the mean connectivity for various soft-threshold powers. **E** Clustered dendrograms were cut at a height of 0.25 to detect and combine similar modules. **F** Dendrogram of all DEGs clustered based on the measurement of dissimilarity (1-TOM). The color band shows the results obtained from the automatic single-block analysis. **G** Clustering dendrogram of module feature genes. **H** Heatmap of the correlation between the module eigengenes and clinical traits of CRC. TOM: topological overlap matrix

**Fig. 6:**
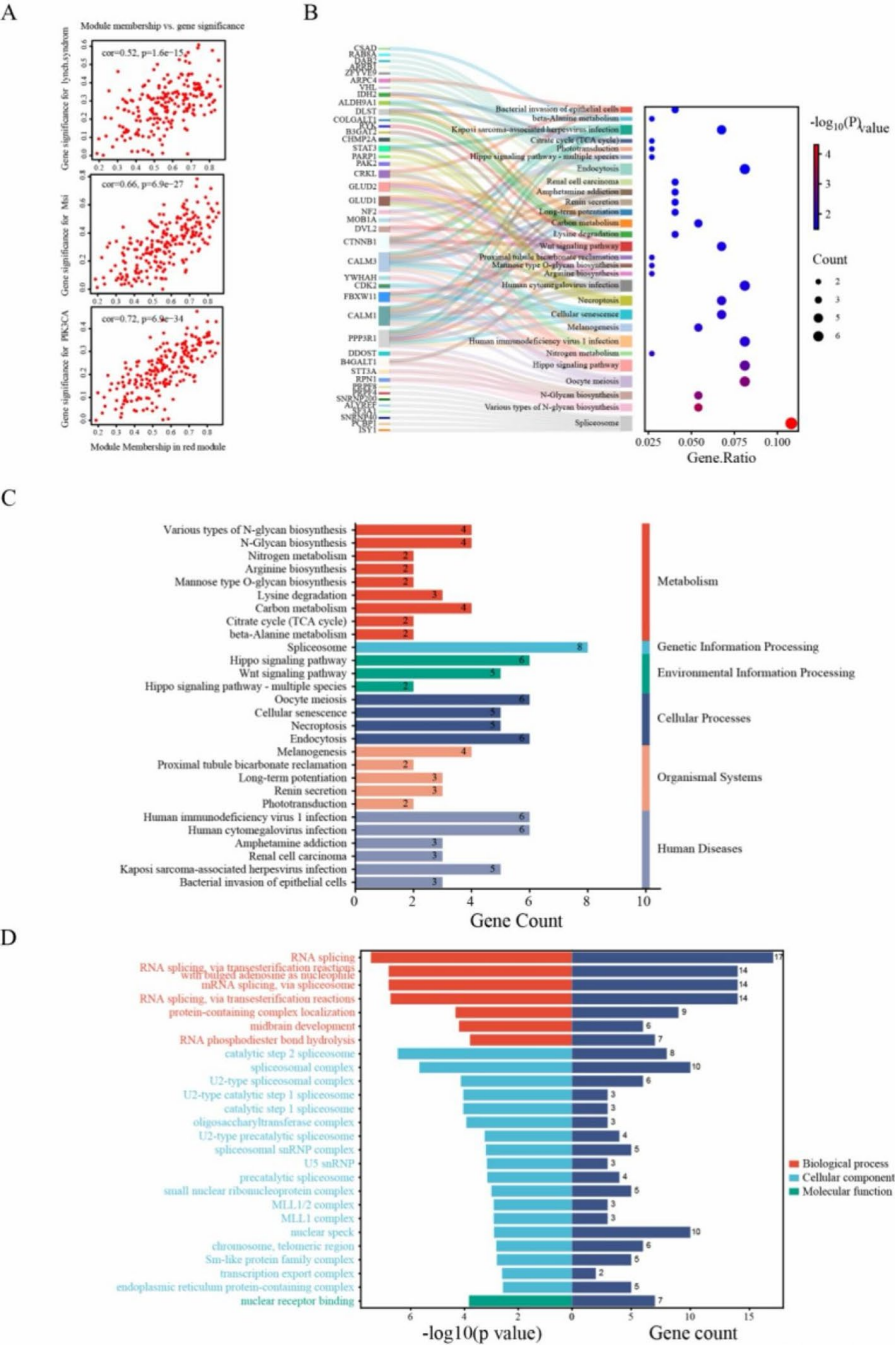
GO and KEGG enrichment analysis of DEGs between EOCRC and LOCRC. **A** Module Membership versus Gene significance scatter plot of lynch syndrom, MSI and PIK3CA. **B** Sankey dot plot for KEGG enrichment analysis of DEGs. **C** KEGG enrichment analysis of DEGs. **D** GO enrichment analysis of DEGs

The GSE39084 dataset comprises a matrix consisting of 21,729 genes. Genes with a adjusted *P* value < 0.05 were deemed statistically significant, resulting in the identification of 2829 DEGs between EOCRC patients and LOCRC group. The volcano plot in Fig. 5A illustrates the overall distribution of DEGs. To further identify the core genes associated with EOCRC more precisely, we employed the WGCNA algorithm to construct a gene co-expression network. The results of sample-level clustering analysis demonstrated well-clustered samples without any significant outliers, as shown in Fig. 5B. The soft threshold was set to six in order to satisfy the scale-free topology of the network, with a corresponding R² value of 0.90 and a high average connectivity (Fig. 5C and D). By applying a clustering height restriction of 0.25 (Fig. 5E), strongly associated modules were merged, resulting in the identification of eight modules for further investigation. Finally, the initialized and merged modules are presented within the clustering tree (Fig. 5F). Transcriptional correlation analysis within modules proved the reliability of the module delineation, showing no substantial association between modules (Fig. 5G). We explored correlations between these modules and clinical features, revealing that age exhibited negative associations with the Black, Brown, Red, and Turquoise modules indicating their significance in EOCRC. Notably, we identified the red module as particularly clinically valuable in EOCRC due to its significant correlation with Lynch syndrome, MSI, and PIK3CA (Fig. 5H). This module comprised of 168 genes. Furthermore, the red module exhibited a significant correlation between MM values and gene significance (GS) of Lynch syndrome, MSI, and PIK3CA (Fig. 6A).

### Functional enrichment analysis of EOCRC related genes

The KEGG database was utilized for pathway analysis of DEGs. As depicted in Fig. 6B, the enriched metabolic pathways encompassed N-Glycan biosynthesis, Nitrogen metabolism, Arginine biosynthesis, Mannose type O-glycan biosynthesis, Lysine degradation, and Carbon metabolism. Figure 6C illustrated the relationship between DEGs and pathways. In the GO enrichment analysis, a total 26 key pathways were involved in cellular components, biological processes and molecular functions (Fig. 6D).

### Integration analyses of the transcriptomic and metabolomic data

**Fig. 7:**
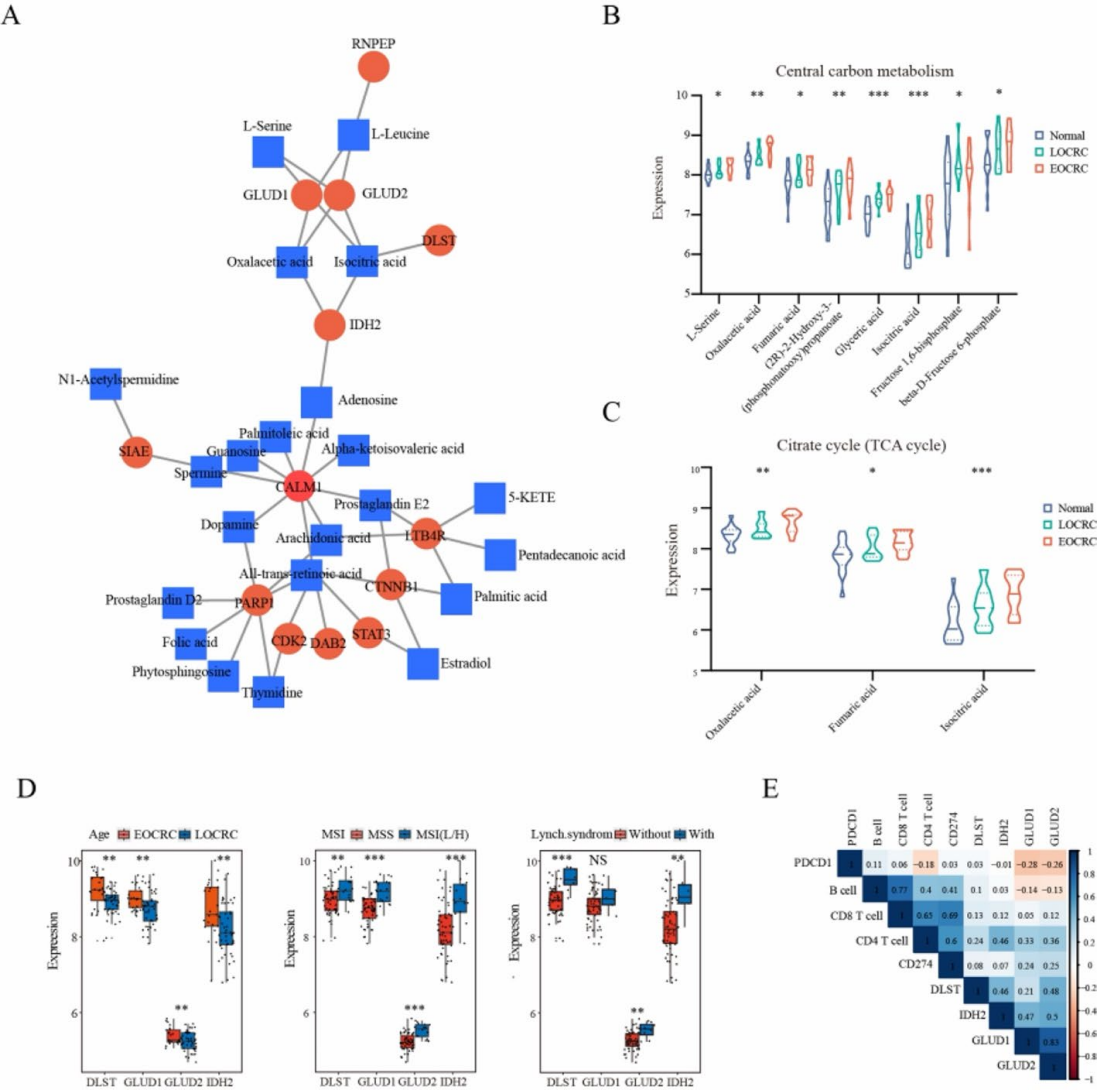
Integrated analysis of metabolomics and transcriptomics screened common metabolic pathways. **A** Co-expression association networks of DEMs (blue nodes) and DEGs (red nodes) were constructed using MetaboAnalyst database. **B** Violin plot of Central carbon cycle related metabolites expressed among the three groups. **C** Violin plot of Citrate cycle (TCA cycle) related metabolites expressed among the three groups. **D** Expression of four genes involved in Central carbon cycle and Citrate cycle (TCA cycle) between different clinical data (age, MSI and Lynch syndrom). **E** Spearman correlation of four genes with immune cells and immune checkpoints (PDCD1 and CD274). *: *P* < 0.05; **: *P* < 0.01; ***: *P* < 0.001; NS: no significance

Pathway-level integrative analysis was conducted using the MetaboAnalyst database to gain a more comprehensive understanding of EOCRC. Figure 7A shows the gene–metabolite network generated based on MetaboAnalyst pathway mapping. Among the enriched metabolic pathways, Central carbon metabolism and Citrate cycle (TCA cycle) were important pathways consistently enriched with DEMs and DEGs. Figure 7B and C show the DEMs of Central carbon metabolism and Citrate cycle (TCA cycle) pathways in EOCRC, LOCRC and normal tissues, respectively. The transcriptome results unveiled DLST, GLUD1, GLUD2, and IDH2 as genes with differential expression in central carbon metabolism and the citric acid cycle (TCA cycle), all of which exhibited high expression levels in EOCRC (Fig. 7D). The expression levels of these genes were positively correlated with each other, suggesting coordinated transcriptional regulation in EOCRC (Fig. 7E). Patients with Lynch syndrome and MSI in CRC exhibit elevated expression levels of DLST, GLUD2, and IDH2. Moreover, these gene expressions demonstrate a positive correlation with CD4 T cells, highlighting their potential relevance for targeted therapy in EOCRC.

## Discussion

The incidence of EOCRC is on the rise, particularly in distal colon and rectal cancers, yet the underlying reasons remain unclear [22]. The current surge in CRC cases among the younger population necessitates further investigation into screening strategies, disease biology, and optimal treatment options specific to this demographic [23]. The presence of metabolic dysregulation was significantly associated with an elevated risk of EOCRC, particularly in cases of proximal and distal colon cancer [24]. Although multi-omics studies have been conducted on EOCRC and LOCRC, the utilization of plasma samples in previous research fails to comprehensively capture the internal metabolic regulation of tumors [17, 25]. Therefore, further research is still warranted.

In this study, we identified distinct metabolic profiles of patients with EOCRC compared to those with LOCRC through tissue untargeted metabolomics analysis. These observed metabolic changes were likely attributed to the dysregulation of multiple metabolic pathways. Transcriptomics analysis revealed novel gene sets associated with Lynch syndrome and MSI. By jointly examining metabolomic and transcriptomic findings at the pathway level, we found that Central carbon metabolism and the Citrate cycle (TCA cycle) were consistently enriched in EOCRC. Because the metabolomic and transcriptomic datasets were obtained from independent cohorts rather than matched samples, this integrative analysis should be interpreted as evidence of pathway-level convergence rather than direct gene–metabolite coupling.

EOCRC are usually diagnosed at a more advanced stage upon detection and have more aggressive histopathology [26]. Approximately 16% of patients with EOCRC were reported to have an associated genetic syndrome, while another 25% have a family history of CRC [27]. However, the specific risk factors for EOCRC have not been fully elucidated, and existing risk models lack sufficient discriminatory power [28].

Hence, one of our primary objectives was to investigate metabolism-based biomarkers for early diagnosis of EOCRC. Here, we conducted LC-MS analysis to identify DEMs between EOCRC and LOCRC. Additionally, we developed a novel prediction model using random forest algorithm that incorporates ten selected metabolites. The model showed encouraging discriminatory ability in this cohort, with a cross-validated AUC of 0.916 for EOCRC. However, it is important to emphasize that this model was developed and evaluated in a single-center cohort with a limited sample size. Although repeated cross-validation was applied, the possibility of overfitting and optimistic performance estimation cannot be excluded. Therefore, the current model should be regarded as exploratory rather than clinically validated. Large-scale, independent, and ideally multicenter studies are required to externally validate the metabolite panel and determine its true diagnostic utility.

Among these hub DEMs, inosine serves as a crucial secondary metabolite and molecular messenger in purine metabolism. Recent studies have revealed that inosine plays a pivotal role as a key regulator of immune checkpoint inhibition (ICI) response across various tumor types [29, 30]. Microbiome-derived inosine has the potential to modulate the response to checkpoint inhibitor immunotherapy [31]. Furthermore, under nutrient starvation conditions, inosine could enhance tumor mitochondrial respiration by inducing Rag GTPases and nascent protein synthesis [32]. Phytosphingosine and Gamma linolenic acid have been reported as potent anticancer agents with efficacy against multiple cancers [33–36]. Formononetin is a natural product with phytoestrogen properties and exhibiting diverse biological functions. It has been demonstrated that formononetin effectively inhibits cancer cell proliferation, invasion, and metastasis by targeting major interconnected signaling pathways. Additionally, it could induce apoptosis and cell cycle arrest through the regulation of intermediary proteins [37, 38]. These findings may provide an explanation for the more favorable survival rates observed among patients with EOCRC despite their advanced stage at diagnosis and histological characteristics [26, 39]. However, the specific roles of other metabolites in cancer were currently limited.

It should also be noted that metabolite annotation in untargeted LC-MS is inherently limited by database coverage and spectral ambiguity. In particular, structurally related or isomeric metabolites may not always be distinguished with complete certainty. Therefore, the biological interpretation of specific metabolites and the associated pathway enrichment results should be considered provisional until confirmed by targeted metabolomics or authentic standards.

Although metabolomics could capture the distinction between EOCRC and LOCRC comprehensively, studying DEGs could further contribute to the development of targeted drugs and determination of therapeutic strategies. Metabolism has an impact on gene expression levels, while genes could regulate metabolic pathways and metabolite content. The combination of metabolomics and transcriptome could provide a more comprehensive understanding of the metabolism and gene regulation mechanism of EOCRC. We employed differential analysis combined with WCCNA to identify the key gene modules, which was associated with Lynch syndrome, MSI, and PIK3CA. Gene enrichment analysis suggested that metabolism-related pathways included N-Glycan biosynthesis, Nitrogen metabolism, Arginine biosynthesis, Mannose type O-glycan biosynthesis, Lysine degradation and Carbon metabolism.

The metabolic pathways that showed significant enrichment analysis included Alpha Linolenic Acid and Linoleic Acid and Arachidonic Acid Metabolism. Previous studies have demonstrated that the peroxidation of n-3 and n-6 polyunsaturated fatty acids in the acidic tumor environment leads to ferroptosis-mediated anticancer effects [40]. Linoleic acid could potentiate CD8 + T cell metabolic fitness and antitumor immunity [41]. The arachidonic acid (AA) pathway plays a key role in cardiovascular biology, inflammatory diseases and carcinogenesis, and can be used as a therapeutic target for tumors including rectal cancer [42, 43]. Previous study has attempted to apply multi-omics using fecal metabolomics and has shown distinct metabolomic and microbiome profiles in EOCRC compared to LOCRC, as well as in subjects without a history of CRC [25]. These profiles include pseudouridine, glycerol, cholesterol, myoinositol, and arachidonic acid. Our study partially aligns with these findings by demonstrating the feasibility of combining tissue metabolomics and tissue transcriptomics through a multi-omics approach. Furthermore, the metabonomics classifier exhibited higher efficiency in diagnosing EOCRC (AUC = 0.916) and LOCRC (AUC = 0.847), respectively.

The integration of metabolomic and transcriptomic data suggested that Central carbon metabolism and TCA cycle were the most significantly enriched pathways in both metabolomics and transcriptomics. By modulating cellular activities, including metabolism and signaling, TCA cycle intermediates are able to impact the processes of cancer development and progression [44]. Targeting TCA cycle function in cancer represents an attractive strategy for cancer treatment, with corresponding clinical trials currently underway [45]. The Central carbon metabolism pathway in cancer involves aerobic glycolysis, increased glutaminolysis, dysregulated TCA cycle, and the pentose phosphate pathway, which is the host’s primary source of energy [46]. In addition to being essential for cancer cell proliferation, Central carbon metabolism also plays a fundamental role in metabolic reprogramming and is vital for endothelial cells, stromal cells, cytotoxic T lymphocytes (CTLs), regulatory T cells, and myeloid cells [47]. Changes of Central carbon metabolism within the cancer stem cell population have been also reported [48].

However, it should be noted that our study has several limitations. First, the small sample size (*n* = 15 per group) may result in sampling errors and increases the risk of overfitting, particularly for machine learning-based modeling. Second, the biomarker panel and candidate genes were not validated in an independent cohort; therefore, the high AUC values, while promising, should be interpreted with caution. Third, metabolomics and transcriptomics were not derived from the same batch of samples, making it challenging to establish precise correlations between metabolites and genes at the individual level. Fourth, our study did not elucidate the specific molecular mechanisms underlying these changes at the gene or metabolite level associated with EOCRC, and functional validation is required. Fifth, metabolite annotation was primarily based on database matching in the untargeted LC-MS workflow, and not all metabolite identities were confirmed using authentic standards. Therefore, some metabolite assignments may remain putative, especially for isomeric compounds, which may affect the interpretation of individual metabolites and pathway enrichment results.

## Conclusions

In summary, our untargeted LC-MS metabolomics analysis revealed unique metabolic alterations specific to EOCRC. The combined analysis of metabolomics and transcriptomics at the pathway level provides valuable insights into the pathogenesis of EOCRC and facilitates the identification of candidate biomarkers and therapeutic targets for further validation.

## Data Availability

The datasets generated and/or analysed during the current study are available in the MetaboLights repository (ID: REQ20251106214462), [[https://www.ebi.ac.uk/metabolights/editor/login](https:/www.ebi.ac.uk/metabolights/editor/login)].

## Abbreviations

EOCRC: Early-onset colorectal cancer
LOCRC: Later-onset colorectal cancer
DEMs: Differentially expressed metabolites
DEGs: Differentially expressed genes
AUC: Area under the curve
HFD: High-fat diet
NMR: Nuclear magnetic resonance spectroscopy
GC-MS: Gas chromatography-mass spectrometry
CE-MS: Capillary electrophoresis-mass spectrometry
LC-MS: Liquid chromatography-mass spectrometry
GEO: Gene Expression Omnibus
OPLS-DA: Orthogonal partial least squares discriminant analysis
WGCNA: Weighted correlation network analysis
MSI: Microsatellite instability
CIMP CpG: Island Methylator Phenotype
GO: Gene Ontology
ROC: Receiver operating characteristic curve
